# Vulnerability in health and social capital: a qualitative analysis by levels of marginalization in Mexico

**DOI:** 10.1186/s12939-020-1138-4

**Published:** 2020-02-10

**Authors:** Oscar A. Martínez-Martínez, Anidelys Rodríguez-Brito

**Affiliations:** Universidad Iberoamericana, México City, Prolongación Paseo de la Reforma 880, Álvaro Obregón, Lomas de Santa Fe, 01219 México City, Mexico

**Keywords:** Qualitative method, Social capital, Marginalization, Support networks, Mexico

## Abstract

**Background:**

Social capital is employed as an asset when there is a lack of an efficient health-care system. However, this relationship is not homogeneous and can differ according to the characteristics of individuals and their context. In this paper, we aim to analyze the role of social capital in the solution of healthcare problems among individuals with different levels of marginalization and unequal access to health services.

**Methods:**

This qualitative study examines the role of social capital in the demand for healthcare among Mexican individuals with different levels of marginalization. The research draws data from semi-structured interviews (*N* = 247) that were collected in four Mexican states with different social welfare benefits: Mexico City, Tamaulipas, the State of Mexico, and Oaxaca. The interviewees were selected using the snowball method and other eligibility criteria such as education, age, and gender.

**Results:**

Findings suggest that social capital is a relevant factor in solving healthcare problems, depending on the level of marginalization. The role of social capital can be explained by the precariousness of medical service delivery, the poor health infrastructure, and the difficult access to health care in Mexico. Networks are the main resource to deal with health related issues, food, medicine, and out-of-the-pocket medical expenses in contexts of high levels of marginalization. In the middle level of marginalization, networks also help in raising funds for more-specialized medical services and higher quality hospitals. In the least-marginalized levels, social capital is used as companionship for sick individuals, while support networks act as emotional relief. At this level, most individuals have private health insurance, and many of them have major medical healthcare coverage.

**Conclusions:**

Participants reported low levels of trust in the health care system because of the poor infrastructure and quality of medical service delivery. Although the main criticism is focused on public healthcare institutions, there is a lack of trust in private medical services as well. These facts are related to the access and quality of medical service delivery and turn social capital into a significant asset. Despite that social bonds or links are valuable resources that individuals can use to solve healthcare related issues, the use of social capital is not homogenous. Indeed, it can be influenced by several factors that were represented in this study through the municipal marginalization index.

## Background

In Mexico, 57.3% of the population do not have access to employment paid health care insurance [1]. This situation represents a scenario of vulnerability, where social capital could play an important role to solve health related issues. To understand the association between social capital and health, it is necessary to understand that the healthcare system conditions and access to healthcare services are marked by deep socio-economic, political, and cultural inequalities. It is also important to note that healthcare includes more than the possibility of getting health-related services. It incorporates access to knowledge about how to navigate the healthcare system, health education, efficient and high-quality medical care, and material and emotional support to deal with illness [2–4].

The National Mexican Council for the Evaluation of the Social Development Policy (CONEVAL) [5] defines the “lack of healthcare access” indicator as the population percentage that does not have healthcare insurance in public or private institutions. The indicator expresses the degree of compliance with the provision of the social right to health access listed in the Mexican Constitution. Although the gap in access to healthcare has decreased in recent years (from 38.4% of the population with no access in 2008 to 16.2% in 2018) [1], the prevalence of Mexicans without access to healthcare coverage is still relevant.

In Mexico, health services are provided by differentiated subsystems [6]. Most of the medical services provided under a healthcare insurance coverage are delivered by the by the Popular Insurance (Seguro Popular), which covers 42.2% of the population who lack of medical insurance as a job benefit given that its beneficiaries do not have permanent job positions. The second most important healthcare provider is the Mexican Institute of Social Security (IMSS), which provides medical services to those employees working in private companies who cover the premiums as a benefit at the formal sector. The recipients of this coverage represent 36.4% of the population. Other healthcare providers are state owned as the Institute of Social Security and Services for State Workers (ISSSTE), which covers 5.5% of the population that receives this benefit as government officials. Other healthcare services are delivered through government managed hospitals who covers benefits to government employees at the National Oil Company (PEMEX), the Ministry of National Defense (SEDENA), the Ministry of Naval Forces (MARINA), state level ISSSTE. Also as healthcare special programs as the IMSS-PROSPERA who provides primary healthcare in low income communities. The remaining population receives medical under the private healthcare insurance, indirect insurance and other medical institutions [7].

Despite the positive trend in healthcare access, Mexico has some of the highest out-of-pocket expenses among all of the nations belonging to the Organization for Economic Co-operation and Development (OECD) [6]. The quality of service and the satisfaction level explain why individuals covered by public institutions prefer to access to private healthcare providers, where there is a larger amount of available facilities (11.4 public hospitals and 28.6 private hospitals for every million inhabitants).

Some of the most common reasons for not demanding services at the public healthcare systems are the long waiting times, the lack of available medications, the distance from the household to health providers, the lack of money, and the poor service delivered by healthcare workers at the clinics [8]. People covered under the Seguro Popular medical insurance reported to have faced more difficulties to receive medical care at its facilities compared to have received assistance at other health systems [9]. This is a remarkable disadvantage considering that most of the beneficiaries of the Seguro Popular are in the first four income deciles and the most economically limited.

Data concerning the conditions of Mexico’s healthcare system suggest a scenario where social capital could play an essential role. Under this premise, we selected four states with differentiated levels of social welfare and healthcare access to conduct our research [10] which can see in Table 1.

**Table 1:** Population Distribution by Type of Coverage (%)

Health-care system	Mexico City	Tamaulipas	State of Mexico	Oaxaca
Seguro Popular	26.4	35.3	44.1	68.9
IMSS	43.0	46.4	35.2	10.4
ISSSTE	12.0	6.4	5.1	5.7
State ISSSTE	0.2	1.3	4.3	0.0
Pemex, SEDENA, and Marina	0.7	1.3	1.0	1.1
Private medical insurance	1.6	0.9	0.7	0.2
Indirect Social Security	2.9	2.4	1.6	0.7
Others	1.0	1.2	0.5	0.1

Source: Authors’ elaboration, based on MCS-ENIGH [7] data

Mexico City (19.6%) and Oaxaca (15.9%) have the highest population percentage without access to healthcare, and are above the national average (15.5%). The State of Mexico has the same percentage than the national level (15.5%), while Tamaulipas has the lowest proportion of individuals without access to healthcare (12.8%) [11].

As it can be seen in Table 1, states with higher marginalization levels (i.e., Tamaulipas and Oaxaca) have more individuals covered by the Seguro Popular and less beneficiares of formal jobs insurance either from private companies or government agencies (i.e., IMSS and ISSSTE). Data suggest a potential association between formal employment, the marginalization index of the state, and the number of individuals covered by the Seguro Popular.

Considering previous statements, we hypothesize that social capital is a relevant asset to manage health problems in Mexico. However, in the case of illness, support varies according to the conditions of marginalization. According to several scholars, families and friend’s social capital can become a crucial option to deal with health issues in the least developed countries where healthcare systems are inadequate [12]. Networks provide assistance, trust, reciprocity and support in case of illness. Therefore, individuals with more social capital are more likely to be healthier [13, 14].

### Social capital and health

Social capital is based on strong and weak ties, cultural dynamics of (re) production, and social articulations, such as a) norms, obligations, and commitments; b) trust; c) expectations of reciprocity; and c) support and cooperation practices [15–19]. Social capital operates at the micro, meso, and macro-social levels [19]. The micro-social level employs networks of relations that can be understood as bonds with relatives and friends and belonging to groups of similar interests [20]. According to Granovetter [17], these networks of relations (or strong bonds) play a decisive role in the solution of particular problems and are the main sources of social and emotional support.

The meso-level refers to the strength of the relations within secondary groups. It comprises neighborhood and the sociocultural institutions of the community as a whole [19]. Therefore, social capital is part of each community’s sociocultural system, its management, and its sanctioning structures. The macro-level includes a feeling of belonging to society and describes the degree of consensus and intensity of social bonds. Besides trust and institutional legitimacy, it includes participation in social, civil, or political organizations [20].

Previous research has reported an association between social capital and health self-perceptions [21–23]. There are also references to the influence of norms and attitudes on healthy behaviors and psychosocial networks that increases access to medical attention and mechanisms that improve self-esteem [24–26]. Social capital is also associated with community participation and mental health [27–29], physical and psychological health [30], and number of years lived [14, 31, 32].

Some authors have described a relationship between informal relations and mental health [33]. In this regard, social capital represents a strategy to mitigate the effects of social isolation and social disconnection on health and quality of life [34, 35]. This is particularly relevant for older adults because it has been found that social capital has positive effects on slowing mental illnesses [36, 37]. Indeed, regular family contacts have positive impacts on loneliness among older adults [38] due to the value of social influence, social support, norms, and the flow of information and resources [39, 40].

In addition, social capital is considered a relevant concept in public health because it helps to identifying the importance of the links or bonds that individuals create throughout their lives [2]. Family, neighborhood, and identity relationships (sources of social capital) shape the source of social control, family support, and creation of benefits through extra-familial networks [41–43]. Several authors have suggested a positive association between social capital and health [44, 45]. Links that individuals build throughout their lives are valuable assets that can be tapped into for personal or community profit [46].

Bonding-type social capital includes trust and cooperation relationships among members of a network that compare themselves as similar because they share a social identity [47]. This form of capital acts as a connector where trust and respect for norms are built, but where intolerance and mistrust toward members outside of the group can also grow. These circles include family members and very close friends [18, 48]. As a consequence, networks are a support system for solving health-related issues [49]. However, behavior that is harmful to health might be reinforced or reproduced because they are ruled by habits, norms, and customs legitimized by the group [17].

Bridge-type social capital includes acquaintances in social groups or networks, where information is shared and exchanged [4, 17, 18]. Regarding access to health care, bridging-type social capital relationships create more opportunities to get information. Individuals can ask friends and other groups to get access to other networks and potentially earn valuable information [50]. Weak links turn into bridges among two or more networks that offer access to information, resources, and health services beyond the intimate circle [51]. The linking-social capital type includes norms concerning respect and trust. It also comprises networks among individuals who interact through different levels of explicit, formal, or institutionalized powers within the society [46, 48].

Several studies show the association between social capital components (e.g., trust and reciprocity) and health depend on the context [4]. The consequences of such a relationship can be observed in populations that are socially excluded, to the detriment, or support to the wellbeing [43]. Social capital highlights the potential of social bonds and interactions, regarding disparities in access to health services [52, 53], and other social, economic, and cultural inequalities [54]. Considering the above, some analyses have included income disparities [12, 54, 55] and the implications of these interactions [2, 56].

However, the positive effects of such associations do not limit the state’s responsibilities concerning public health and social welfare [57]. Social capital is used as an asset in the absence of an efficient health-care system. Nevertheless, the association is not homogeneous and can differ according to the characteristics of individuals and the context [4], mostly in populations that are socially excluded [43]. For this reason, our purpose is to answer, what role does social capital play when people with different levels of marginalization and access to health services get sick in Mexico?

## Methods

### Municipal marginalization index

This study employs the Municipal Marginalization Index [58] as a measure of socio-economic comparison among contexts. The index includes four areas with nine types of exclusion, which are measured by percentages of the population that have no access to basic services. The four areas and nine types of exclusion are (1) education (i.e., illiteracy, population that has not finished elementary school); (2) housing (i.e., homes with no plumbing or sanitary services; homes with no electricity; homes with no water pipes; homes in any level of overcrowding; homes with dirt floors); (3) population distribution (i.e., towns with fewer than 5000 inhabitants); and (4) income (i.e., working population that earns up to two times the minimum average wage). Each of these categories has an indicator expressed as a type of deficiency, which is used to create an index that classifies marginalization in five levels: very high, high, medium, low, and very low [59].

In comparison to other similar measures as the Human Development Index (HDI), the Municipal Marginalization Index represents conditions of high marginalization as more vulnerable because they accumulate the highest percentages of indicators. Therefore, in low marginalization populations, there is a very low proportion of exclusion indicators. We labeled the marginalization values as follows: I (very low), II (low), III (medium), IV (high), and V (very high).

### Sample

Data were collected through semi-structured interviews in four states with different degrees of social wellbeing [10]: Mexico City (very high level), Tamaulipas (high level), the State of Mexico (medium level), and Oaxaca (low level). Interviews were carried out in different municipalities according to the type of municipality (urban, semi-urban, or rural), Municipal Marginalization Index (IMM) [59], and population size (see Table 2). The total number of municipalities selected was 71 and distributed in the following way: 16 in Mexico City, 6 in Tamaulipas, 31 in the State of Mexico, and 18 in Oaxaca.

**Table 2:** Sociodemographic Characteristics

Level of Marginalization					
	Very low	Low	Medium	High	Very high
Type of Municipality	Urban: 85.5% Semi-urban: 14.5%	Urban: 79.3% Semi-urban: 15.5% Rural: 5.2%	Urban: 76.5% Semi-urban: 2.9% Rural: 20.6%	Urban: 50.9% Semi-urban: 12.3% Rural: 36.8%	Urban: 81.8% Rural: 18.2%
Population at that level of marginalization (%)	30.8%	23.5%	13.8%	23.1%	8.9%
Education level					
	Elementary: 11.8%	Elementary: 27.6%	Elementary: 14.7%	Elementary: 28.1%	Elementary: 13.6%
	Secondary: 18.4%	Secondary: 17.2%	Secondary: 20.6%	Secondary: 21.1%	Secondary: 22.7%
	High School: 27.6%	High School: 19.0%	High School: 26.5%	High School: 17.5%	High School: 36.4%
	College: 31.6%	College: 29.3%	College: 26.5%	College: 29.8%	College: 22.7%
	Graduate: 10.5%	Graduate: 6.9%	Graduate: 11.8%	Graduate: 3.5%	Graduate: 4.5%
Age					
	Mean: 44.86	Mean: 46.75	Mean: 43.21	Mean: 44.02	Mean: 43.45
	Range: 20–74	Range: 17–87	Range: 25–64	Range: 23–87	Range:25–72
Gender					
	Men: 48.7%	Men: 53.4%	Men: 50%	Men: 47.4%	Men: 40.9%
	Women: 51.3%	Women: 46.6%	Women: 50%	Women: 52.6%	Women: 59.1%

Source: Authors’ elaboration

A total of 247 semi-structured interviews were conducted: 78 in Mexico City, 44 in Tamaulipas, 53 in the State of Mexico, and 72 in Oaxaca. Interviewees were chosen through non-probabilistic sampling considering variables as socioeconomic level, educational level, age, and gender to achieve heterogeneous profiles (see Table 2). According to previous research [60], interviewees with different socio-demographic profiles support saturation of categories, which produces more sound and consistent results.

Access to the communities was done with the help of several gatekeepers who were residents in the area. They were the starting point to the snowball technique to find individuals with the desired profiles.

### Information gathering

Interviewers were trained in ethical research issues, as well as in the use of the protocol to handle respondents’ information. Every interviewee was informed that all of their responses would be confidential and agreed to sign informed consent. The interviews lasted approximately thirty minutes major the social capital and health section. Answers were recorded with the interviewees’ permission and later transcribed literally on a text processor.

### Analysis

Data analysis was iteratively deductive and inductive, based on the grounded theory [61] that lies on fieldwork as the main knowledge source to come to conclusions based on empirical evidence [62].

Coding was concurrent with data analysis and interpretation. First, it helped to observe, to record, and to classify answers during fieldwork. Second, coding helped to disaggregate information and to group and to synthesize it from multiple associations and cognitive inferences [62]. This procedure was helpful to the interview analysis at different stages, such as understanding, synthesizing, and theorizing phases. The understanding stage enabled us to approach to the beliefs, values, ​​and ways of life of the different contexts and to see the experience from the participants’ perspective [63].

During the synthesis stage, we read the transcripts of the interviews. The purpose was to identify and codify the emerging trends in each category. To that end, we used the qualitative NVivo software, version 11. The following codes emerged from this process:

1. Accessibility to healthcare. Service location and type of medical service.
  1.1. Quality of care, infrastructure, and provision of medicines.
  1.2. Confidence in health institutions and medical staff.
2. Reasons for not requesting medical services.
  2.1. Alternatives to feel better.
3. Self-perception of social security and health conditions.
4. Networks and belonging.
  4.1. People with whom I would count in accident situations, health problems, and the need for attention of sick family members.
    4.1.1. Expected support types.
    4.1.2. Associated feelings.
  4.2. Other support networks or groups.
    4.2.1. Types of support expected from other networks.
5. People who would help in accident situations, health problems, and the need for attention of sick family members.
  5.1. Expected support types.
  5.2. Associated feelings.

In general, this codebook guided us to clarify the association between social capital and health. In specific, the codebook was also helpful to distinguish central discourses and meanings associated with the role of social capital in health, according to the informants’ levels of marginalization and access to health services.

Finally, the theorizing stage allowed us to contextualize and to explain the relationship [63]. Authors discussed the findings in several meetings. Interpretations of categories in each level of marginalization and their link with the theoretical framework were examined during these meetings.

## Results

### Municipalities and interviewees

In this study, municipalities were grouped into levels of marginalization and type (i.e., urban, semi-urban, or rural municipality), as shown in Table 2.

Table 2 shows a potential relationship between the municipal marginalization level and the interviewees’ socioeconomic profile. Data suggest most of the individuals with elementary school education attainment, in very low and low marginalization areas, live in rural and semi-urban areas. In contrast, those participants in areas with very high and high levels of marginalization with undergraduate and graduate studies attainment, live in urban areas.

### Social capital and health issues

Regardless of the level of marginalization, social capital represents an essential tool to deal health issues for the interviewees. Low trust in health institutions turns social capital into a critical component to optimize the individual’s economic resources. Bridge-type social capital contributes to the choice of medical service providers, by obtaining information from previous experiences and recommendations from neighbors, friends, and family. Moreover, interviewees indicated that belonging to networks of friends, relatives, or neighbors provides information that allows a better choice of hospitals, specialist doctors, and administrative procedures regarding medical insurers. Regardless of the marginalization level, findings show that the exchange and dissemination of health information are central resources for the closest networks.

Emotional support is also fundamental to face and solve health-related problems. Still, there are some differences in how it works because it depends on the level of marginalization and access to health services.

Participants living in very low or low marginalization areas, have health coverage in 96.5% of the cases. Such coverage includes access to private health care providers (e.g., health insurance with full coverage), as well as access to specialist doctors. At this level, participants have access to healthcare coverage due to their membership to a larger number of networks which include extended family, workgroups, or religious groups, besides strong links (bonding-type social capital). The interviewees’ socio-economic level and their health coverage influence the function of social capital. In this case, it provides an emotional support system that leads to feelings of security and support. For instance, people accompany or visit a sick person: they give them fruit, they accompany them to the doctor, or they help with the housework.

*I feel, very, very supported. I mean I’ve known my friends for a long time, and I have no doubt that they would help because, at least with what we have shared, that’s how they make you feel, don’t they? And concerning my relatives, well, the time we spend together when I see them, um, let’s say that they make me feel sure that, that they would give a hand whenever it might be needed, right? And … well, economically I’d do it because I have the insurance that would cover all their expenses* (man, 36, IMM I)*.*

Individuals that live in high or very high marginalization areas are covered mainly by the Seguro Popular or lack of healthcare insurance. Seguro Popular has limited coverage of services, quality of the infrastructure and care, provision of medicines, and very high waiting times. For these reasons, 72.9% of interviewees mentioned that they had “insufficient infrastructure, for the number of individuals covered.” Regarding funding for medicines, 54.5% of respondents stated they rely on generic medications, which affects the quality of the treatment. Meanwhile, 75.6% of respondents indicated to be unsatisfied with the quality and access to health services. Therefore, they would prefer to use private doctors’ services (at low cost walk-in clinics where generic drugs are prescribed), home remedies, self-medication, traditional therapies, praying, or drinking. When they do not have enough money, respondents pray with the aim of tolerating the pain or healing.

At these levels of marginalization, relatives and friends networks are valuable assets that individuals can use to transform into alternative and additional resources to solve health issues. Bonding-type social capital integrates the material and emotional support to deal with health situations. In the case of accidents or illnesses, these networks are useful to raise funds for the patient’s treatment. Likewise, networks provide necessary emotional support to improve the health of a sick person.

*My family. Well, my brothers and my parents. I feel that in a strong need, yes, I have support from them. We have the support of the whole family* (man, 49 IMM IV).

These individuals’ socio-economic situation and their vulnerable position regarding health coverage emphasize the essential role of social capital. According to our findings, support networks establish a critical resource for health emergencies, such as access to medicines, home remedies, or to transfer the patient to the nearest health care center.

*We have helped each other in the good and the bad times... if someone passes away or get ill, we collect money, and we help* (woman, 47, IMM IV).

*To my friends and family, I would help them with actions ... because I do not have much money* (woman, 23, IMM V).

Participants who have experienced these situations expressed they felt supported and grateful. Simultaneously, they felt guilty for being a burden to their loved ones with such moral and material responsibility. They also expressed constant feelings of vulnerability.

*Well, [I feel] very, very bad, because I can’t even find a way to pay for it so that I can take care of it by myself or take care of my own accidents or illnesses, so you feel bad. I think anyone feels bad, don’t they? That someone has to pay his/her expenses, and not out of pride, but because to have to say I can’t pay it by myself, I mean it’s hard, isn’t it?* (man, 31, IMM IV)

The micro-level of social capital is essential in high or very high marginalization levels because networks provide the patient with information to navigate the healtcare system and to have accurate information about doctors and alternative health care centers. Likewise, people in networks recommend home remedies to the patient or donate medications that they did not consume in previous illnesses. To respondents, the support of these networks facilitates the transportation of the sick person to the health centers. In emergencies, private cars are a frequent alternative to the lack of ambulance and medical emergency services.

In the middle level of marginalization, respondents present heterogeneous healthcare in coverage and how social capital works in different ways in case of illness. On the one hand, for individuals who have major medical health insurance, social capital is similar to very low and low levels of marginalization in situations of illness. On the other, for those who have the services of IMSS, ISSSTE, and Popular Insurance, and do not have major medical health insurance, social capital works similarly to those interviewed at high and very high levels of marginalization.

Finally, 51% of respondents indicated that they have a low level of trust in health institutions. Although the main criticism targets at public institutions, individuals also mistrust the private ones. Results suggest that, regardless of the marginalization level, there are specific codes about the support levels, the type of support, and its length in Mexico. In the case of disease or long term health problems, the commitment level and the possibilities of material and emotional support are stronger in the family nucleus. Regarding friends’ and neighbors’ networks, commitment and support are usually temporary.

## Discussion

The most vulnerable socio-economic levels are the most affected in the access to health services. Behind these issues, there are deficits in health sub-systems. For instance, there is a reduced number of doctors and nurses in the system (i.e., 2.2 doctors and 2.6 nurses for every 1000 inhabitants), including those that work in the private sector [6]. In addition, the public system usually provides less time for each patient’s visit, which has negative consequences on the quality of medical service delivery. Also, only some of the Seguro Popular, IMSS, and ISSSTE medical centers have emergency rooms [6]. Finally, 33% of prescriptions in the Seguro Popular were not wholly fulfilled because the medicine was not in stock, while at the IMSS, 14% of the prescribed drugs were not available [6].

Regardless of the municipal marginalization level and the healthcare access, health system conditions could explain why most respondents mentioned that they do not use the basic or emergency services from public institutions. Most individuals indicated that these services are not efficient or effective. This perception is the result of (1) sick individuals that must travel long distances to reach the health care centers, (2) long waiting times, (3) perception of poor quality medical services delivery, and (4) there is a lack of funding for prescribed medications.

Disparities between levels of marginalization suggest another source of inequality. Findings evidence that the participants at the poorest condition are in a very vulnerable situation regarding their right to health. Disparities in access to and quality of health services, explains the role of social capital to address health related problems. The evidence here supports that the micro-level of social capital has an essential role when individuals get sick. However, unlike other studies [63], the relevance of social capital can be explained by the precariousness of medical services, the poor health infrastructure, and the problems of access to medical care [6, 8]. The low levels of trust in physicians and health institutions make bridging-type social capital an essential component to select a good quality medical service according to the individual’s budget. However, the respondents’ socioeconomic level and health coverage influence the role of social capital, or in other words, the type of support they provide and receive.

Each social capital type has different functions. The bridging-type social capital helps to provide information about physicians or homemade remedies. Bonding-type supports emotional and instrumental provision to manage health problems [25, 51]. Regardless of the level of marginalization and interviewees’ socio-economic level, bridging-type social capital provides information based on direct experiences. These networks help in search and choice of healthcare insurance, economical and reliable hospitals, medical specialists, and advice for the follow-up and care of diseases [25].

Findings also evidence the value of emotional support in health situations [64, 65]. In the low and very low marginalization levels, support networks are broad and include strong links (e.g., close friends and family), religious groups, and extended family. In the very high and high levels of marginalization, networks are limited to the nuclear family and closest friends. In specific, respondents in very high and high levels of marginalization indicated that family, close friends, and neighborhood are essential to face accidents or severe illnesses. These networks not only provide the necessary emotional support for the recovery of the patient, but they also articulate instrumental support [51, 66], namely medicines, homemade remedies, taking the patient to the nearest hospital, and funding for health services.

At medium marginalization level, social capital is broader: most of the participants mentioned they could count on extended family and friends. Networks also help in raising funds for more extensive medical services and better hospitals. For individuals in the low and very low marginalization levels, support networks include strong bonds that also extend to other groups and act as emotional support. In addition, the links are broader, and a positive relationship between social capital and income is developed [66]. Considering most of the participants at this last level have private healthcare coverage, social capital operates mostly as emotional support.

## Conclusions

Mexican Constitution guarantees that every person hast the right to health protection. Health is a social right. According to interviewees, the delivery of the healthcare services is deficient, mainly at the primary care level as well as at the emergency room services provided by the state operated hospitals, as the Seguro Popular, IMSS, and ISSSTE. The access to this right should not be conditioned by any socio-economic, political, and cultural inequalities.

In Mexico, employment status (formal or informal employment) and socio-economic level define the access to the health care system and subsystems. The IMSS and ISSSTE relate to formal employees in the private sector and the federal government, respectively. Meanwhile, the Seguro Popular focuses on individuals in the informal sector not covered by the benefits of a formal job or permanent position medical insurance. Evidence suggests a positive association between informal work, socioeconomic vulnerability, and access to healthcare services. The population proportion covered by the Seguro Popular seems to be associated both to the municipality’s informal employment level and its marginalization index.

Participants indicated low levels of trust in healthcare institutions due to the poor infrastructure and quality of services. Although the main criticism is focused on public institutions, individuals also did not report to not trust the private ones either. These facts related to the access and quality of medical services and transform social capital into a significant asset. Besides, strong bonds or links are valuable resources that individuals can tap to solve health-related issues; however, the use of such resources is not homogenous and is modified by specific factors that were represented in this study through the municipal marginalization index.

We can observe that at low and at very low levels of marginalization, the interviewees’ socio-economic conditions and health care coverage (which in many cases includes major medical insurance) impacts on the role of social capital. Social capital acts as a type of emotional support and as a way of companionship that explains the positive feelings of security and support. At high and very high marginalization levels, networks are limited to the nuclear family and the closest friends. These individuals’ access to healthcare defines social capital as an essential resource and an emotional support system when dealing with medical issues. For this reason, participants reported contradictory feelings. On the one hand, they feel supported and grateful, but on the other, they feel guilty about burdening their loved ones with such moral and material responsibility.

One of the implications of this study is that, in Mexico, the use of social capital (especially bridging and linking types) could be a valuable asset in health education, access to information, and health resources and services. Besides, it could help to foster norms for respect and trust between medical healthcare providers and patients as a sine qua non for the quality and efficiency of health services. The second implication of this study is that, although we found that social capital is an essential resource for solving healthcare issues, the positive effects of the relationship do not exonerate the responsibility of the Mexican government to guarantee the quality of the healthcare services provided by the government. We should emphasize the importance of ensuring access to healthcare and quality medical services. It means to reduce waiting times, to increase prescription medicines availability, and having a more balanced ratio of medical staff in comparison to the number of individuals covered.

Finally, there are some limitations to this study. First, the IMM is an indicator that provides measures at the context level conditions (neighborhood, municipality and, state) and not at the individual level (or by household). Second, the highest percentage of people interviewed were located in urban areas. Even in the locations under study, there are no rural areas at the very low level of marginalization (see Table 2). Third, the results are not generalizable to the entire Mexican population given that a qualitative analysis approach was used. Despite the sample cannot be considered as representative, the selection of the interviewees considered the heterogeneity and diversity of socio-economic, cultural, and geospatial aspects of the participants in order to improve learning possibilities and the reliability of the results. In this regard, the coding procedure used allowed the incorporation of emerging nuances and interpretations, which contributed to the consistency of the findings.

Despite these limitations, the qualitative approach used in our research can be appropriate to any context (region or country) where health services have been stratified according to population socioeconomic conditions or employment status. It could also be useful in those countries where non-contributory health services cover individuals living in poverty because it shows how functional social capital can be in health-related situations.

## Abbreviations

CONEVAL: National Council for the Evaluation of Social Development Policy
HDI: Human Development Index
IMM: Municipal Marginalization Index
IMSS: Mexican Social Security Institute
ISSSTE: Institute of Social Security and Services of State Workers
OECD: Organization for Economic Co-operation and Development
